# Barriers to and facilitators of diabetes self-management practices in Rupandehi, Nepal- multiple stakeholders’ perspective

**DOI:** 10.1186/s12889-021-11308-4

**Published:** 2021-06-29

**Authors:** Mandira Adhikari, Hridaya Raj Devkota, Tomris Cesuroglu

**Affiliations:** 1 Nepal Development Society, Bharatpur, Nepal; 2 Institute for Social and Environmental Research-Nepal, Pokhara, Nepal; 3 Faculty of Science, Vrije University, Amsterdam, The Netherlands

**Keywords:** Self-management, Diabetes, Barriers, Facilitators, Nepal

## Abstract

**Background:**

Self-management of diabetes is associated with glycaemic control and adherence to medication and healthy lifestyle practices. There is lack of information on the barriers to and facilitators of diabetes self-management practices in low income country, Nepal. This study aimed to explore the barriers to and facilitators of Type 2 diabetes self-management practices taking multiple stakeholders’ perspectives in Nepal.

**Methods:**

Four focus group discussions and 16 semi-structured interviews with people with Type 2 diabetes, caregivers, health care providers and health managers were conducted from April to May 2018 in Rupandehi district of Western Nepal. They were audio-recorded, transcribed, and analysed using a thematic approach.

**Results:**

Five main themes emerged that influenced diabetes self-management practices: individual factors, socio-cultural and economic factors, health system and policy factors, availability and accessibility of resources, and environmental factors. The important barriers were: lack of knowledge about diabetes self-management practices, cultural practices, insufficient counselling, lack of guidelines and protocols for counselling, and financial problems. The major facilitators were: motivation; support from family, peers, and doctors; and availability of resources in the community.

**Conclusion:**

Based on our findings, a multilevel approach is needed to address these barriers and facilitators. These findings will help guide strategies to develop programs that impart knowledge and skills to improve the diabetes self-management practices of people with Type 2 diabetes.

**Supplementary Information:**

The online version contains supplementary material available at 10.1186/s12889-021-11308-4.

## Background

The increasing prevalence of Type 2 diabetes has become a major global health challenge. According to the International Diabetes Federation, one in every 11 adults are living with diabetes [1]. Diabetes prevalence has been increasing steadily over the past decades particularly in the low and middle-income countries. Nearly 86.4 million people are living with Type 2 diabetes in South Asia alone in 2014 [2]. In Nepal, there is a strong heterogeneity in diabetes prevalence across the studies (range: 6.3 to 23.5% [3–6]) and between rural (1.03 and 2.5%) and urban areas (8.1 and 14.6%) [4, 7]. The prevalence of diabetes (pooled-prevalence: 8.5, 95% CI 6.9,10.4%) is further expected to increase due to rapid urbanization and lifestyle alternations [8].

Despite the magnitude of the burden in Nepal, knowledge, attitude and practice of patients with Type 2 diabetes on the management of diabetes is poor [9, 10]. Early diagnosis, treatment and self-management are essential for the prevention and control of the disease. Successful management of diabetes also requires people with diabetes to adhere to self-management practices. Self-management is a process of developing knowledge and skills to manage the complex nature of diabetes in the social context [11]. The International Diabetes Federation [12] has emphasized the importance of self-management in controlling diabetes. Self-management of diabetes requires people with diabetes to follow certain behavioural actions consistently on a daily basis. These actions are monitoring blood glucose level, following a diet plan, maintaining foot care guidelines, engaging in physical activities, and taking medications either in the form of insulin or oral medications as indicated [13, 14].

The literature on diabetes across the globe reports that diabetes self-management is difficult [15–19]. The studies on diabetes self-management practices draw attention to different factors that are barriers to or facilitators of self-management of diabetes, and these factors also vary according to the settings. Factors that play as barriers to diabetes self-management practices were: difficulty in adjustments to lifestyle after being diagnosed with diabetes [17, 19]; the lack of knowledge [18]; the lack of culturally relevant knowledge [20]; and not recognising the importance of self-management practices [18]. Further, communication barriers exist both from the people with diabetes and health providers’ side. The patient level communication barriers are trust issues with the health providers on the self-management counselling, technical language used in communication while health providers level of communication barriers include lack of time to discuss self-care strategies and lack of psychosocial support to people with diabetes [21]. Different factors such as access to physical activity facilities [20], financial issues [22] and practicing alternative medicines [9, 23] were identified as barriers to self-management of diabetes. Diabetes self-management facilitators were acceptance of diabetes diagnosis [17]; support from family and health care providers [16, 17, 19]; and availability of counselling classes on Type 2 diabetes [16]. Sociocultural factors were highlighted as both barriers and enabling factors to self-management [24–29]. Sociocultural factors such as use of alternative therapies, priority to family needs, misconceptions that diabetes is a curable disease are the major barriers for diabetes self-management [24]. Further, locally ingrained cultural and lifestyle practices and non-compliance to diabetes self-management practices are other challenges to diabetes self-management [24, 29]. Support from family and community in terms of emotional support and availability of time to buy medicines, cooking food and accompanying to visit health facilities enable people with diabetes to self-manage their diabetes [27, 28].

An extensive search of literature revealed that only two quantitative [30, 31], one mix-method [32], and some qualitative studies [33–35] investigated the barriers and facilitators of diabetes self-management practices in Nepal. These studies explored barriers and facilitators of some components of diabetes self-management practices, while other components such as blood glucose monitoring and foot care were not explored. A quantitative study from central Nepal conducted by Kadariya and Aro explored the barriers to and facilitators for the diabetes self-management particular to physical exercise. The authors identified barriers to stay active were lack of time due to family affairs such as household chores and time for children [31]. Bhandari et al., [32] found that people with diabetes prefer to go to work than doing physical exercises. A multilevel approach qualitative study identified lack of knowledge on physical exercises and laziness as main reasons for non-compliance with physical exercise recommendations [33]. On the other hand, facilitators for physical exercise were experiencing better health due to improved sleep, reduced level of fatigue and increased mental alertness [31]. Additionally, supportive peers enabled people to stay physically active. Religious belief was another facilitator to engage in physical activities. For example, people more frequently visit temples by walking, which helps to keep them physically active [32].

Sapkota et al., explored the impact of food and food culture on the dietary compliance of Nepalese people with diabetes in Nepal and Australia [35]. Lack of knowledge about diabetes diet is barrier for people with diabetes [30, 33]. Negative perception on the food intake was reported as a barrier. For example, people with diabetes perceive that they need adequate amount of food to counteract with diabetes medication [35]. Further, pressure from relatives and friends in social gatherings to eat unhealthy foods was an issue faced by people with diabetes to comply with dietary recommendations [35]. Lack of option for healthy food in the markets and lack of guidelines on dietary management were barriers to dietary compliance [33]. The other qualitative study explored the influencing factors to adhere to diabetes medication among people with diabetes in Australia and Nepal. The authors reported that the inadequate understanding about the importance of diabetes medication to maintain blood glucose level. People with diabetes discontinued medication after the blood glucose level was within controlled limit [33, 34]. Financial barrier was reported in the studies where people with diabetes had to compromise other needs to buy medicines [33, 34]. Financial constraints can force individuals to use natural methods for blood glucose control and discourage people with diabetes to initiate diabetes medication. Lack of year-round availability of medicines can further hinder diabetes self-management [36]. A multilevel study identified lack of access to health facilities to regularly monitor blood glucose level was a barrier for people with diabetes living in rural areas and lack of money was a problem to comply with medication recommendation and regular blood glucose monitoring in rural Nepal [33]. Limited study explored barriers and facilitators of diabetes self-management practices from the perspective of various stakeholders in multiple levels [33]. No studies covered comprehensive understanding of the issues related to diabetes self-management practices (monitoring blood glucose level, following a diet plan, maintaining foot care guidelines, engaging in physical activities, and taking medications), presenting a significant gap in the literature. The present qualitative study aimed to gain deeper insights on the barriers to and facilitators of diabetes self-management practices in Nepal from the perspectives of people with type 2 diabetes, caregivers, medical doctors, district health managers, and a social worker.

Involving different stakeholders in the study provides an opportunity to explore the self-management of diabetes from different viewpoints and to develop programs specific recommendations for diabetes self-management. Stakeholders participation is based on the assumption that each stakeholder has a specific perspective on a particular issue and the involvement of different stakeholders will result in better understating of the problems, solutions and help to take better decision to improve the health status of community people [16]. Involvement of stakeholders in health research is recognised as strengthening the scientific approach and increasing the chance of implementation of health interventions [16]. In this study, stakeholders play a role only as participants; because of time constraints stakeholders could not participate in the topics of research, composition of sample and recruitment methods, development of data collection tools, analysis and dissemination of findings.

## Methods

### Study setting

The study was conducted in Rupandehi district of Nepal, a district that is on Nepal’s southern borders with India. The population of this district in 2012 was approximately 880,000 with 51% of the population aged 20 or above [37]. The human development index of Rupandehi district in 2014 was 0.498, higher than the national index (0.490) [38].

In Rupandehi, two referral government hospitals are located in urban areas. Public health services in rural areas are provided through five primary health care centres, 64 health posts and one District Public Health Office [39]. Medical officers (MBBS) are responsible to provide primary care including diabetes in hospitals and primary health care centres. Nurses’ roles include mainly for the provision of inpatient care in hospitals and maternal and child health services in the primary health care centres. Health assistants (Diploma in General Medicine) and auxiliary health assistants (Community Medical Assistant) provide primary care in health posts. Many patients go to pharmacies for medical care. These pharmacies are unregulated therefore, the quality of care from pharmacies and those who work there is questionable. Additionally, there is non-existence of diabetes (self-management) groups in the hospitals and primary health care centres in Rupandehi.

There is no available information for the prevalence and risk factors of diabetes, and self-management practices in the Rupandehi district. We purposively selected Lumbini zonal hospital and Motipur primary health care centre (PHC) for this study. The facilities were chosen from the list of public facilities that were currently providing diabetes management services provided through trained medical doctors and laboratory facilities to ensure accurate diabetes diagnosis.

### Study design and participants

This was a qualitative exploratory study with the inclusion of multiple stakeholders who were involved in the diabetes self-management practices in various roles: people with Type 2 diabetes, caregivers, medical doctors, district health managers and a social worker. Qualitative study design focuses on understanding of social world through listening to participants viewpoints [40]. Further, information obtained from the explorative study is rich in content and values experiential knowledge of participants [41]. Focus group discussions (FGD) and semi-structured interviews (SSIs) were data collection tools in this study. In the FGD, participants are encouraged to talk to each other, share views and comment on each other’s experiences. The assumption of FGD is the group dynamics can encourage people to share and confirm their views in a way that would be less feasible in a one to one interview [42, 43]. The SSI is widely used qualitative data collection method in health research [44] and is conducted with the use of a guide that contains open-ended questions on a topic to be explored by the researcher. The guide helps to keep the interview focused and to explore a research topic more systematically and comprehensively [44, 45].

The people with type 2 diabetes in this study refer to the patients that were registered as patients with type 2 diabetes of the Lumbini zonal hospital and Motipur PHC. The caregivers refer to the family member/spouse/relatives who provided support for diabetic patients at home. The health care providers refer to medical doctors that were involved in providing diabetes care in Lumbini zonal hospital and Motipur PHC. The zonal hospital is a tertiary level hospital and covers the patient load of surrounding districts as well. The district health managers refers to the professional who worked as public health officer level in the district public health office. The social worker refers to the professional who worked in one of the non-governmental organisations in the Rupandehi district.

This study followed Bronfenbrenner’s ecological system theory [46] and Whittemore et al.’s conceptual framework [47] based on social ecological model which is used to explain the prevention and management of diabetes. According to this model, to change the behaviour of individuals for the long term, the programs must target at multiple level of influences. The level of influences are categorised into intrapersonal factors, interpersonal, institutional, community and public policy level [46–48].

Intrapersonal factors that shape individual’s health behaviours are attitude, self-efficacy, and communication skills [49]. Individual’s attitude, and confidence influence the ability to change the behaviours to improve diabetes self-management practices [50]. A patient’s interpersonal relationships are within a social context such as family, friends, neighbours [51] and health professionals [52]. The support from a social context is beneficial to improve diabetes self-management practices [53]. The forms of social support are categorised into companionship, emotional, tangible and informational support [54]. The institutional influence on individual include work, school and religious settings. Institutional factors can provide a context to promote healthy behaviours and diabetes prevention activities to a large group of people, thus forming a social support, which further facilitates healthy behaviour adoption. An individual’s community factor consists of geographical area where he/she lives. The characteristics of the neighbourhood and communities have an influential role for health behaviour of individuals [47]. Public policy influences health behaviours of individuals through the implementation of laws and policies by local and national authorities.

### Focus group discussions with people with Type 2 diabetes

Participants were included for focus group discussions if they were of more than 20 years of age, diagnosed with Type 2 diabetes for more than a year prior to the FGDs, and registered either in a PHC or a Lumbini zonal hospital. They were recruited using a purposive sampling method who meet the inclusion criteria. Participants within focus groups were mixed however, they were not selected based on stratification such as age, gender or time since diagnosis.

The information of people with Type 2 diabetes on age, sex, duration of diagnosis and address was obtained from outpatient registers of the PHC and hospital. Then, a researcher contacted people with Type 2 diabetes through phone calls and explained the aim of this study. Participants who agreed verbally over the phone were included in FGDs. In addition, written informed consent was obtained prior to FGDs. The primary researcher was the moderator for FGDs and a trained nurse was an observer of the FGDs. The observer had also participated in pretesting of tools as well and was responsible for note taking. The moderator was responsible for managing the group interactions so that everyone would get opportunity to express their view and the FGD was not dominated by a few. Four FGDs were conducted with 26 participants ranging from six to eight in a group. The number of FGDs were determined by saturation of information as sample size of qualitative research cannot be predetermined [55].

Semi-structured interviews were conducted with caregivers, medical doctors, district health managers and a social worker. Caregivers were selected for semi-structured interviews (SSIs) if they had assisted people with Type 2 diabetes (as a family member) for more than a year prior to the interviews. They were recruited with the help of people with Type 2 diabetes. Caregiver and people with Type 2 diabetes were matched but they were interviewed separately. For example, information from the people with Type 2 diabetes was obtained from FGD while information from the caregivers was obtained from SSIs. Then, a researcher contacted caregivers through phone calls and explained the aim of this study. Five interviews were conducted until data saturation reached [56].

Medical doctors who worked as general practitioners in the outpatient department of Motipur PHC and Lumbini zonal hospital were included due to their experience of Type 2 diabetes management. They were contacted directly by the primary researcher, and seven interviews were conducted. It was not possible to recruit diabetes specialists, because there were only few diabetes specialists in the country and no specialist in the district.

District health managers were recruited and interviewed through direct contact by the primary researcher. This study included a public health administrator, a public health officer and a focal person for non-communicable diseases (NCDs) from the District Public Health Office. Three interviews were conducted with these participants as these three officials were responsible for the coordination and management of public health services for NCD in Rupandehi [39].

One interview was conducted with a social worker from a local non-governmental organisation, who has community level experience of working with disadvantaged and vulnerable people and was recruited with the help of a local public health researcher.

The findings on barriers to and facilitators of diabetes self-management practices were strengthened by triangulating the data from FGDs and SSIs, and comparing and contrasting the viewpoints of different stakeholder groups [57].

### Data collection

Data collection was carried out between April and mid-May 2018. The FGDs were conducted in a room inside a community hall; the SSIs with medical doctors, district health managers and a social worker were conducted in a closed room in the workplace of the participants; the semi-structured interviews with caregivers were conducted in a room in participants’ homes. The duration of each FGD ranged from 60 to 90 min, while each SSI ranged from 30 to 45 min.

The design of the FGD and SSI guides were based on Whittemore et al.’s [47] conceptual framework for applying the social ecological model to the prevention and management of diabetes. The framework covers intrapersonal, interpersonal, institutional, community and public policy factors suggested by Bronfenbrenner’s ecological theory [46].

Topics covered in the FGDs and SSIs were:

- barriers to diabetes self-management practices: intrapersonal (knowledge, motivation, responsibility), interpersonal (relationship with family, friends/peers, health professionals, and neighbours), institutional (health system factors), community (cultural values, availability and accessibility of resources for diabetes self-management practices) and public policy factors (diabetes self-management practices policies and funding)
- facilitators of diabetes self-management practices: intrapersonal (knowledge, motivation), interpersonal (relationship with family, friends/peers, health professionals, and neighbours), institutional (health system factors), community (cultural values, availability and accessibility of resources for diabetes self-management practices) and public policy factors (diabetes self-management practices policies and funding)

The topic guides were pre-tested in a similar setting and adjusted on the basis of participants' feedback. Minor changes included the order of questions and rewording some questions. First, selection of another ward adjacent to the study area was determined for the focus group and interview with care givers. The recruitment of people with Type 2 diabetes was done by the local health worker. Then, participants were informed about date and time of FGD. The recruitment of the caregivers was done with the help of participants of focus group discussion as it is feasible to identify caregivers due to time limitation. Pretesting of SSI interview guides with the medical doctors was done with a medical doctor from another district. Due to time unavailability, pretesting was not possible with the district health managers.

The tools were translated into Nepali and back translated to English by the first author (MA). Once finalised by the first author, the co-author HRD who is also a native speaker checked language clarity and finalised tools. The final English version of the tools are attached as supplementary files.

Data collection was conducted in the Nepali language by the first author. FGDs and SSIs were audio-recorded to facilitate data analysis. A trained nursing graduate assisted the first author in all FGDs and interviews by taking charge of audio-recording and taking notes.

### Data analysis

FGD and SSI were transcribed from the audio recordings and focus group notes. A thematic inductive approach was utilised to analyse data as suggested by Braun and Clark [58]. First, transcripts were read multiple times to become familiar with the data. Then, the data was coded by the first author on atlas.ti software and all the patterns were checked to ensure they were relevant to the research questions [58]. Coded data with similar meaning were grouped together to form sub-themes, which were reviewed multiple times by the author and co-authors. Themes were then finalised by merging common sub-themes. Connections between different themes and sub-themes were displayed using thematic maps. Finally, these themes were checked again to ensure they addressed the research questions, relevant quotations were selected to illustrate the findings.

### Ethics

Ethical approval was received from the Nepal Health Research Council (registration number 72/2018). The Human Research Ethics Guideline of this council follows the declaration of Helsinki declaration for research involving human subjects [59]. Written and verbal informed consent was obtained from each participant before their participation. Anonymity and confidentiality of the information was maintained by removing personal identifiers from the data. The notes and audio tapes are kept in secured password protected electronic device accessible only to the first author and the co-authors.

## Result

This study identified wide range of barriers to and facilitators of Type 2 diabetes self-management practices. The findings are presented based on themes according to the level of socio-ecological model. They were: individual level factors (with sub-themes of knowledge, motivational factors, responsibility, beliefs and time constraints); interpersonal level factors (sub-themes consisted of social network, doctor-patient relationship); community level factors (sub-themes: cultural values, availability and accessibility of resources, environmental factors and media); organisational level factor with a subtheme of multidisciplinary team; and policy level factors (sub-themes: guidelines and polices, and economic factors). The themes are presented separately, but barriers and facilitators at each level are influenced by multiple factors across at least the three levels.

Consistent information from different groups of participants was observed. People with Type 2 diabetes expressed their experiences, and caregivers talked about their experiences of providing care to people with Type 2 diabetes at home. Additionally, doctors described patients with poorly managed diabetes and other factors that have an influence on diabetes self-management. District health managers and a social worker expressed their opinions on the factors influencing diabetes self-management mainly from the health system and policy factors.

### Individual level factors

#### Knowledge of diabetes self-management strategies

Knowledge about diabetes self-management practices was reported as a barrier and a facilitator of diabetes self-management practices. Participants (doctors and people with Type 2 diabetes) described a lack of knowledge about a healthy diet, medication initiation and adherence and foot care, along with confusion about blood glucose monitoring tests as a barrier for self-management. Some of the people with Type 2 diabetes reported that blood glucose monitoring is not needed if they are compliant with medication.

***“****We do not know that diabetes patients must care about feet. We do not know how to look after our feet… We have no knowledge about it, so we haven’t looked after our feet.” –* (Person with Type 2 diabetes, male)

*“Living with diabetes means you need to eat small portions frequently. But in reality, many diabetes people eat too much at one time. It is due to lack of knowledge on healthy eating.”-* (Person with Type 2 diabetes, female).

*“Talking about Nepalese context- there is a perception of diabetes patient that if I miss one dose of medicine, there won’t be any harm on me…they said I forget to take medicine today. But it is not they forget to take it. It is about lack of understanding of the importance of taking medicine regularly”. -* (Medical doctor, male)

*“Diabetes people are confused on the types of blood tests done to monitor blood glucose level. We do fasting and post prandial blood test to monitor blood glucose level. If we prescribe for fasting blood test and inform patients that come to the health facility without meal, but patients come to the facility after meal. They need to come another day to get test done. There is due to lack of knowledge about the importance of blood glucose monitoring”. -* (Medical doctor, male)

*“I take medication every day, so I don’t need to do regular blood glucose monitoring”. -* (Person with Type 2 diabetes, male)

Many participants (people with Type 2 diabetes and medical doctors) mentioned that knowledge of diabetes self-management practices enabled people with diabetes to follow recommended diabetes self-management practices.

*“I must take diabetes medicines; patients must know that they have to take medication daily. I am taking medicine regularly in the morning and evening. I know I have to consume medicine for my diabetes.”* – (Person with Type 2 diabetes, female)

*“I know that I need to do some physical exercise daily to control blood glucose level and I always walk or engage in other physical activities.”- (*Person with Type 2 diabetes, male*)*

*“One helping factor for diabetes patient to monitor blood glucose regularly is the knowledge. Diabetes patients who are aware of doing regular blood glucose monitoring always come to health facility to have blood glucose level checked.”- (*Medical doctor, male)

#### Motivational factors for diabetes self-management practices

Motivation was an important enabler to initiate and continue diabetes self-management practices, whereas lack of motivation was a barrier. Carelessness was also reported as a barrier. Most people with Type 2 diabetes were motivated to self-manage however some of them were not motivated to engage in self-management. Medical doctors linked that motivation is related to lack of understanding the importance of doing self-management strategies.

*“I know the consequences of not doing exercise regularly …I have no motivation to do exercise early in the morning. This laziness is killing me every single day…I can’t explain how much I have no motivation to do other self-management practices as well.” (*Person with Type 2 diabetes, female)

*“Many patients seem motivated once we tell about the importance of engaging in self-management. I think lack of understanding of the need to be compliant with self-management strategies is somehow related to motivation to initiate and continue with self-management.”* - (Medical doctor, male)

Participants such as people with Type 2 diabetes, doctors and caregivers stated that motivated people with Type 2 diabetes adhered to diabetes self-management practices. Motivations included: to live healthy, to prolong life with diabetes, to avoid the consequences of complications, and to control diabetes. The sources of motivation were peers, family members, and doctors. The ways of getting motivated by peers is through sharing of experiences with diabetes self-management at home.

*“If diabetes patient has motivation, it helps a lot to do diabetes self-management practices”-* (Caregiver, male)

*“(..) If diabetes patient is concerned about diabetes… if he has a feeling of getting better, then he shows readiness to do the things to control his diabetes. In my case, “I am motivated to control my diabetes and I do not want to experience complications”- I am ready to do whatever I have to do in order to control my diabetes. I am managing time to do physical exercise.” – (*Person with Type 2 diabetes, female**)**

*“I want to manage my diabetes, so I go to do regular blood glucose monitoring test and take medication daily. I want to live long without experiencing complications; I really want to control my diabetes.”- (*Person with Type 2 diabetes, male**)**

*“I have a very supportive family. They always motivate me to stay active, encourage me to take medication and healthy diet. I have a good friendship with my friends who are diabetic as well. We encourage each other to control diabetes by sharing our experiences.”-* (Person with Type 2 diabetes, male**)**

*“My doctor always encourages me to manage my diabetes at home in each visit. I feel motivated do my best to control diabetes after a visit to a doctor.”-* (Person with Type 2 diabetes, female**)**

#### Responsibility for diabetes self-management practices

People with Type 2 diabetes were perceived as being in charge of their health by patients and caregivers. Being responsible towards own health enabled them to manage diabetes, which is facilitated by the knowledge on diabetes self-management practices and support from family.

*“Diabetes patients must control on what they can eat and what they can’t. Getting sick from eating unhealthy food and go to a doctor the other day is not wise. Patient must be responsible to control their diabetes”- (*Person with Type 2 diabetes, male)

*“Diabetes patients themselves look after their feet. It is the patient who is in-charge of their body. I think feeling of responsibility towards their own health helps to manage the diabetes”- (*Caregiver, female)

*“Feeling of responsibility to manage diabetes must come from the inner heart. If the patient is concerned about their health and wanted to manage diabetes, he is ready to do everything. I am ready to do anything to control my diabetes. I am managing my time sometimes in the morning and sometimes in the afternoon for physical exercise. Also, I think being knowledgeable on the self-management strategies also helps to manage diabetes effectively.”-* (Person with Type 2 diabetes, female)

*“Patients themselves to some extent are responsible to control or worsen diabetes. Sometimes the support and cooperation from the family is helpful for the patients to do right things to manage diabetes at home. In my case, I always support my mother’s actions to manage diabetes*.” – (Caregiver, male)

#### Beliefs

Few people with Type 2 diabetes and medical doctors stated beliefs on the alternative medicines were barriers for medication initiation and adherence. Examples of alternative medicines from the people with Type 2 diabetes side was topical use of plant leaves on foot sole and consumption of some herbs and use of ayurvedic medications. Having a belief and practicing alternative medications was influenced by neighbours and people in the community.

*“Patients focus on traditional ways of medicine. They believe in ayurvedic medicine and consume it. Some of the diabetes patients come to us with complications, we provide them information on the medication and other self-care strategies, but they do not listen to what we told them. When they go to their community, neighbours and other people told them the opposite of what we said. They believe these people and keep practicing traditional medicines.” –* (Medical doctor, male)

*“I heard from some people in my community that herbs cure diabetes. There is one type of plant (Calotropis gigantea); the leaf of the plant is believed to lower blood glucose level when the leaves are kept on the sole for overnight for a few days. I tried this for three days, and I did not take medication these days.”- (*Person with Type 2 diabetes, male)

*“After I was diagnosed with diabetes, I did not take diabetes medication. I consumed soaked fenugreek seeks for a month with a belief to control blood glucose level. But it did not work, and I again went to a doctor. After visiting a doctor, I have started to consume medication daily.”-* (Person with Type 2 diabetes, female)

#### Time constrains

Many participants from all groups stated that the time factor is a barrier for women to stay active and do regular blood glucose monitoring. They have limited time for themselves to stay healthy. It is due to family commitments such as looking after kids and other family members. Medical doctors stated that time management to monitor blood glucose levels is difficult for working patients. Most of the time, people with Type 2 diabetes had to take a day off to come to the health facility for blood glucose monitoring. People with Type 2 diabetes who were labourers had a choice to come to a health facility or go to work to feed their family.

*“I am struggling to find time to do my daily exercise. I have so many things to do at home for example, doing household works, looking after kids and many more.”-* (Person with Type 2 diabetes, female)

*“Women do not have time to do exercise. They are busy doing household chores from the morning till night. They really struggle to find a suitable time for exercise and go to health facilities for blood glucose monitoring. Also, labour workforce is affected by time constraints. If they choose to come to a health facility, they had to leave work for that day resulting loss of daily wages to run a family.”-* (Medical doctor, male)

### Interpersonal factors

#### Social network: family, neighbours and peers

Participants such as people with Type 2 diabetes and their caregivers stated that lack of family support to manage diabetes is a barrier for diabetes self-management. They further cited that female people with Type 2 diabetes had less support from their family to maintain healthy diet and comply with physical exercise requirements. Another illustration of their difficulty to maintain healthy diet was unavailability of diabetic meal at home.

*“A family is a barrier for regular physical exercise … particularly to the female diabetes patients. Even though she has the motivation to do physical exercise, she can’t do it as she has to look after children, prepare meals … when will she get time for physical exercise?” – (*Caregiver, female)

*“There is a compulsion for diabetes patients to eat what is cooked for other healthy family members because they want to eat any types of food. It is not possible to ask family members to eat diabetic diet every day.”* – (Person with Type 2 diabetes, male)

Several doctors mentioned that negative influence from neighbours was reported as a barrier to continue medication intake and physical exercise. Neighbours created confusion by telling people with Type 2 diabetes that medication had negative effects and expressed concerns over their daily physical exercise. These interference from neighbours plays a barrier to comply with diabetes self-management practices.

*“There is an influence of neighbours … They tell diabetes patients that once you start taking your medicines you cannot discontinue it … patients trust on their words and they do not want to take medicine.”-* (Medical doctor, male)

*“When patients walk for physical exercise, neighbours ask them- where are you going? Why are you going? Patients had to answer them each time and they feel demotivated to stay active.” –* (Medical doctor, male)

Many participants reported the supportive roles of families (spouse, children, and daughters-in-law), peers/friends, neighbours and self-help groups to manage diabetes. For example, caregiver (son) motivated their parents provided time and financial resources to manage diabetes at home. In addition, people with Type 2 diabetes were encouraged to comply with a healthy diet when families shared the same diet, so that the people with Type 2 diabetes do not have to feel he/she is having diabetic diet.

*“I get a lot of support from my family (..) apart from emotional support, my son always gives me money to buy medicines and sometimes he bought medicine from pharmacy. The support I get from my family motivates me to manage diabetes. (…) In our family, we eat same food, so I don’t feel I am eating diabetic diet. I feel lucky to have a caring family*.” (Person with Type 2 diabetes, male).

The other support from family and friends/peers were to remind people with Type 2 diabetes to take medicine on time, carry out blood glucose monitoring tests, maintain foot care, and continue physical exercise. This supportive role was facilitated by knowledge on diabetes management and information sharing between people with Type 2 diabetes and family members. Family members and peers/friends accompanied people with Type 2 diabetes to health facilities for follow-up visits and kept them company when exercising. Such support from family members, and the sharing of experiences among people with Type 2 diabetes, informed and motivated patients to sustain diabetes self-management practices.

*“Family help me when I have to visit a doctor. It really helps me. Sometimes they also go with me and sometimes they help me to reach health facility.”- (*Person with Type 2 diabetes, female)

*“Family provides support in different areas. They can tell diabetes patients not to eat unhealthy food, take your medicine daily, go to health facility for regular blood glucose monitoring, when you are going somewhere, bring your medicine with you…these kind of suggestions from family can be helpful for diabetes patients.” (*Focal person NCD, male)

*“Peers provide information on foot care. They told me that,” I should not be careless on my foot care”. -* (Person with Type 2 diabetes, male)

Some people with Type 2 diabetes stated the positive role of neighbours to arrange transportation to travel to a health facility when family members were not at home, purchase vegetables from a market when people with Type 2 diabetes could not go and prepare sugar free meals for social events.

*“If I have to go to some one’s place, they provide me sugar free snacks. There is much help from neighbours.”-* (Person with Type 2 diabetes, male)

#### Doctor- people with Type 2 diabetes relationship

The relationship between doctor and people with Type 2 diabetes influences diabetes self-management practices positively and negatively. Some participants such as doctors and district health managers discussed the lack of comprehensive counselling, and continuity of counselling, during a doctor’s appointment. Limited time for counselling was a barrier for doctors to provide counselling because of their high load of patients. Doctors’ lack of knowledge about diet planning was a barrier to providing counselling on a culturally appropriate healthy diet. Doctors were also unlikely to offer advice on foot care.

*“Service providers do not give counselling to diabetes patients on diabetes self-management practices because of lack of time … they just write medicines. Patients are not provided enough information as they need.”- (*Medical doctor, male)

*“Doctors in the public health facilities have high patient load (..) Comprehensive counselling is needed to educate patients living with diabetes as they have to care for different aspects of diabetes management such as healthy eating, concordance with medication and physical exercise recommendation and other areas. Also, counselling to the patient is needed on all aspects of diabetes self-care in each visit to a doctor. However, doctors cannot provide detailed counselling on all aspects of diabetes self-management strategies due to lack of time and high patient load.”* - (Public health officer, male)

Doctors were regarded as an enabling source of information about key areas of diabetes self-management practices such as the importance, dosage and timing of medication; and the benefits of exercise to maintain blood glucose levels. Many participants particularly people with Type 2 diabetes were not aware of about other forms of physical activity such as yoga and bicycle riding, and people with Type 2 diabetes believed that other forms of exercise would help them to do regular physical exercise. Information from the doctors about foot care and healthy eating were helpful in two ways for some of the patients. First, the people with Type 2 diabetes applied the information in their diabetes self-management practices; second, they shared the information received from the doctor with family members. Knowledge sharing with family members helped create a supportive environment for diabetes self-management practices at home.

*“Doctors provide counselling to their patients and that is how patients understands the importance of medicine.” –* (Caregiver, female)

*“Diabetes education is helpful. If we, doctors, can provide detailed information to diabetes patients on when to take medicine, what is the duration of medicine intake, information on continuity of medicine, it helps. It is helpful for diabetes patients to continue taking medicine.”-* (Medical doctor, male)

Good communication between doctors and people with Type 2 diabetes was another reported facilitator of diabetes self-management practices. Open communication was vital for both doctors and people with Type 2 diabetes to understand a patient’s issues related to diabetes self-management practices, and to advise related solutions.

*“Doctors should tell diabetes patients on medicine: why it is needed? ... Doctor’s advice is extremely useful for patients to continue taking medicine.”-* (Caregiver, male)

*“Diabetes patients interact with doctor without fear; this helps to ask questions when in confusion. Good relationship between patient and doctor is helpful to manage diabetes.” (*Person with Type 2 diabetes, male)

### Community level factors

#### Cultural values

Most participants mentioned negative and positive cultural influences on dietary practices and medication compliance. People with Type 2 diabetes and caregivers reported dietary misconceptions influenced them to eat only certain vegetables, fruits and cereals. The other barrier to maintaining a consistent healthy diet reported by several participants were food habits, such as a preference for a carbohydrate diet and bulky meal in the evening, and a craving for sweets Also, many participants cited that food preparation method, for instance use of a lot of oil, spices and overcooked food were barriers to eat healthy food. Doctors stressed that consuming carbohydrate-based meal limits intake of other nutrients.

*“After my diabetes diagnosis, I have completely avoided potatoes and some other vegetables, rice, and fruits because friends said diabetes people can’t eat these vegetables and fruits.”-* (Person with Type 2 diabetes, male)

*“The way we cook and consume food is not healthy for patients with diabetes. We have a tradition of cooking food in a lot of oil and spices which is not considered healthy these days. Also, we have a habit of consuming bulky food in the dinner.” –* (Medical doctor, male)

*“Cultural factors are intertwined with eating healthy meal. Generally, we do not care about eating balanced and healthy diet. We give preference to carbohydrate rich food and we eat bigger portion of it in each meal.” -* (Public health officer, male)

Social events such as festivals and social gatherings were seen as an excuse to eat unhealthy food, and encouragement or pressure from family members or peers to eat unhealthy food at social events presented further barriers. At times, when healthy food was unavailable at social events, it difficult for people with Type 2 diabetes to socialise.

*“We have different festivals and other social events such as weddings. Some of the diabetes patients have a feeling of eating food that they are not supposed to eat. They take these events as an opportunity to eat food they’ve been eating before they had diabetes.”-* (Social worker, female)

The concept of preparing different meals for people with Type 2 diabetes and other members of a family has started to change. Knowledge about the benefits of healthy eating motivated family members to adopt a healthy cooking style with less oil and fewer spices. Participants cited the availability of suitable food helped them to enjoy festivals, religious and cultural events. Catering for the dietary needs of people with Type 2 diabetes was facilitated by family members and peers having more knowledge about healthy diets for diabetics.

*“We have adopted ourselves to healthy eating practices that is suitable for my father-in-law. We, family, are being very supportive to our father-in-law.”-* (Caregiver, female)

*“People in the community are aware of diabetes ... they are aware of diabetes diet. I have no problem to eat healthy food during social functions.”-* (Person with Type 2 diabetes, female)

#### Availability and accessibility of resources

Resource availability and accessibility influenced diabetes self-management practices for people with type diabetes. For example, diabetes self-management barriers included: the unavailability of suitable food at the market, home, and restaurant; unavailability of space for physical exercise in a community; and unavailability of year-round medications in health facilities.

*“Some of the diabetes patients do not have healthy food to eat at home … in this case, they eat what they have without considering the nutritional value they need for. They have no choice to eat healthy options.”-* (Medical doctor, male)

*“We do not have sugar free and high fibre food at home all the time. We have to go to market which is far from our community. Sometimes that is a problem to eat healthy diet for diabetes patients”-* (Caregiver, female)

*“We have lack of open space or other options for exercise.”-* (Medical doctor, male)

Many participants including doctors and people with Type 2 diabetes cited that availability of healthy food options relates to the purchasing capacity of people with Type 2 diabetes. Many doctors mentioned that recommended visits to a doctor could not be made when the health facility was far away from the community. This is particularly barrier for the people with Type 2 diabetes living in rural areas. The other concern from doctors was the prescribing and dispensing medicine by a non-licenced practitioner and without proper counselling to the patients. This practice acts as a barrier for the people with Type 2 diabetes to understand the importance of medication and respond to side effects.

*“Unavailability of healthy food choices in the community is one barrier. The capacity to buy healthy food is the underlying factor for people to afford healthy food available in the markets.”* (Medical doctor, male)

*“Transportation issue is another concern for patients living with diabetes to regularly visit health facilities to do blood glucose monitoring. Patients from remote areas have to walk whole day to go to primary health centre just to check blood glucose level*.” – (Medical doctor, male)

*“In private pharmacies the wife of a doctor, who is not a doctor or authentic person gives medicine. She doesn’t know how to do counselling to the patients on the side effects and other relevant information.”- (*Medical doctor, male)

When resources were available, people with Type 2 diabetes were enabled to adhere to recommended diabetes self-management practices. For example, the availability of suitable food near health facilities helped people with Type 2 diabetes to enjoy healthy meals during a visit to a doctor, and private pharmacies improved year-around availability of medicines. Similarly, shorter distances between home and health facility enabled people with Type 2 diabetes to easily see a doctor. People with Type 2 diabetes were encouraged and motivated to follow recommended diabetes self-management practices when they had access to health facilities.

*“There is a provision of diabetic diet in the restaurant near the health facility where I go to see my doctor. Money is quite expensive but I am happy that I can eat healthy food. I found it very easy to go to doctor because of availability of diabetes-friendly food.”-* (Person with Type 2 diabetes, female)

*“We can buy medicines at nearby private pharmacies. Sometimes I bought medicines from the private pharmacy when I realise when I have no medicine left. I do not bother about the cost in this kind of emergency.”*- (Person with Type 2 diabetes, male)

*“If there is availability of blood glucose monitoring facilities in local health institutions such as health posts, it is easier for patients to regularly monitor blood glucose level. (..) patients also feel encouraged to maintain self-management strategies.”* - (Medical doctor, male)

#### Environmental factors

Environmental factors negatively influenced people with Type 2 diabetes management of diabetes. Participants such as public health officers and social worker stated that increasing urbanisation has limited areas for recreation and exercise, and increased pollution, which are barriers for physical exercise in urban areas. Medical doctors also added that bad weather such as rain, landslide and humid were the deterrents for meeting physical exercise goals and medication adherence. People with Type 2 diabetes living in hilly region cannot buy medicines during monsoon due to non-availability of transportation.

*“In cities there is rapid urbanisation that results in increase pollution level. Diabetes patients do not like to walk in the streets with inhalation of dust particles every single day.”- (*Social worker, female)

*“Diabetes patients stay at home and do less exercise because of pollution they face while walking on road.”*- (Public health officer, male)

*“Weather condition plays a barrier to become concordant with exercise. During summer the temperature outside is very hot that restricts to do physical exercise.”-* (Medical doctor, male)

*“*People *living in hilly areas struggle to come to health facilities to buy medicines during rainy season. In the rainy season, there are few transports available or sometimes there is none in case of flooding or landslide.”* – (Medical doctor, male)

### Policy level factors

#### Guidelines and policies

Many doctors and public health officers highlighted the lack of guidelines and policies as barriers to diabetes self-management practices. They discussed the difficulties of providing counselling on diabetes self-management practices because Nepali-specific diabetes self-management counselling guidelines and protocols were unavailable. Such guidelines should incorporate the types of food available in the community and physical exercise methods appropriate to the Nepalese community. In addition, there was no policy to increase the capacity of health facilities such as availability of laboratory tests in the health post and sub-health post levels. Lack of such policies put pressure on doctors, because there was less time available to provide the required counselling.

Furthermore, the lack of policy regarding free medication and free blood glucose monitoring tests limited people with Type 2 diabetes to the medication and blood glucose monitoring tests that were recommended by doctors who, without the benefit of policy and clinical guidelines, rely on their own knowledge or experience. Such practice only resulted in inconsistent and variable advice being given to people with Type 2 diabetes.

*“You know, we have to follow Western guidelines, but we are not Western … where are the guidelines suitable for Nepali context and culture?”-* (Medical doctor, male)

“*We do not have any policies on diabetes self-management. It is the main barrier. We give emphasis on communicable diseases.” –* (Medical doctor, male)

### Economic factors

Economic circumstances were cited only as a barrier to diabetes self-management by all participants. However, physical exercise was the only diabetes self-management practice which was not affected by participant’s economic status as walking did not cost money. Lack of funds to attend health facilities, buy medication and conduct regular blood monitoring tests was a barrier to people with Type 2 diabetes. In addition, people with Type 2 diabetes who struggled with low financial resources, could not buy vegetables and other foods, and appropriate footwear. A lack of money prevented people with Type 2 diabetes from following diabetes self-management practices despite being motivated to do so.

*“The biggest problem is money. If I don’t have money, how can I buy medicine? How can I arrange for healthy foods? And how can I go to health facility, and do blood glucose monitoring?”. -* (Person with Type 2 diabetes, female)

*“I cannot always comply with medication adherence. Sometimes I had no money left to buy medicines”*. - (Person with Type 2 diabetes, male)

*“It costs to buy medicines and check blood glucose level. On top of that, transportation cost is a burden for patients to go to health facilities. Those who have money issues cannot do timely visits to a doctor.”-* (Public health officer, male)

*“Some patients do not comply with medicine intake and regular monitoring of blood glucose level due to lack of money.”-* (Caregiver, female)

*“Money matters to stay compliant with diabetes self-management strategies for patients with diabetes. For example, even patients with diabetes are aware of the benefits of healthy diet, that will not solve the issue of healthy eating. If there is lack of money how can patients afford healthy diet?”*- (Medical doctor, male)

## Discussion

This qualitative study explored a wide range of barriers and facilitators to improve diabetes self-management practices from perspectives of people with Type 2 diabetes, caregivers, medical doctors, district health managers and a social worker. At individual level, knowledge, motivation, responsibility, beliefs and time constrains were the influencing factors for people with Type 2 diabetes to manage diabetes at home.

### Individual level

Our study highlighted a lack of knowledge as an important barrier to diabetes self-management practices, in line with previous review articles [60, 61]. A qualitative study from South Asia supports our finding that lack of knowledge hindered people with diabetes to practice foot care [62]. Further, a review article from India found people with diabetes were unable to follow recommended medication and dietary guidelines, because of low health literacy about disease and its self-management [63]. However, a review study from United States argued that knowledge is not sufficient to carry out diabetes self-management practices. It stresses the social support from the family and friends, and access to health services also influence people’s ability to carry out diabetes management [64].

Motivation was a facilitator of diabetes self-management practices. For example, motivation to stay healthy facilitated maintenance of physical exercise, and friends and peers were the sources of motivation to continue healthy lifestyle habits. These findings are supported by previous work from Nepal, which shows that people with diabetes feel encouraged to stay physically active when they are supported by peers [31]. The other facilitating factor in diabetes self-management practices was taking responsibility for one’s own health.

Consistent with a previous study from Korea, facilitator of diabetes self-management practices among people with diabetes was a patient’s feeling of responsibility towards their own body [65]. Responsible people are motivated to learn in-depth information about disease and its management. In our study feeling of responsible towards diabetes self-management was influenced by the knowledge on the diabetes management strategies, which is supported by the previous study [66]. Past studies have described the complex relationship between diabetes responsibility and motivation in relation to self-efficacy for management of the disease. A study from South Korea identified that patient’s feeling of responsibility towards their own health was crucial for diabetes self-management [65]. Responsible people were motivated to learn in-depth about the disease and its management. Our study further expanded this finding by showing that feeling of responsibility towards diabetes self-management was influenced by the knowledge on the diabetes self-management strategies [66]. The feeling of responsibility, confidence and the ability to manage their health were associated with diabetes self-management [67, 68]. Particularly, being confident in their actions and having a sense of self-efficacy made people with diabetes responsible for their health [69]. To illustrate, people with diabetes who were confident in their diet plans and medication regimens had controlled glycaemic levels [69]. Conversely, a lower sense of self-efficacy negatively impacts people’s accountability to make changes needed to manage their disease. For instance, individuals blamed themselves for not adhering to self-management recommendations when a glycaemic level was not maintained [70]. People with diabetes who feel the responsibility were taking appropriate actions to manage diabetes. For instance, responsible people were concordant with medication recommendations [71]. Our study only shows that people with diabetes feel responsible for self-management of the diabetes in daily basis; further studies should explore the complex relationship between diabetes responsibility and self-efficacy in relation to management of the diabetes in community-based settings. Previous studies have highlighted the relationship between motivation and responsibility of own health and diabetes outcomes [72, 73]. Motivated people are more likely to adopt healthy lifestyles and become active to and feel responsible for the outcomes that were resulted from their behaviours [73]. There is some evidence that diabetes self-efficacy is correlated with self-management of the disease (e.g. confidence towards diet, exercise and medical treatment). A study from Nepal showed that people with diabetes having high level of self-efficacy were also confident, and were able to initiate and maintain physical exercise activities [30]. A study from Oman further linked diabetes self-efficacy with self-management practices including following healthy diet, engaging in physical activities, and regular blood glucose monitoring [66]. Combined with diabetes self-efficacy, adequacy of diabetes (medication) regimen and adherence, and compliance to the regimen is crucial for diabetes self-management and needs vigilant attention in community-based settings [69].

Time constrains was emerged as the barrier to manage diabetes at home. This was particularly applicable for the women. Further, women living with diabetes in our study received less support to manage diabetes. Lack of support from family puts burden on women to look after themselves and dependents such as children. In the patriarchal society like Nepal, women are expected to do household chores, looking after children and elderly [31]. These responsibilities limits women to manage time to do physical exercise and visit to a doctor.

### Interpersonal level

At the interpersonal level, family, peers and neighbour either play a supportive role or become barrier to manage diabetes at home for people with diabetes. Consistent with previous findings [31], lack of support from close social contacts including family and friends was a barrier to physical exercise. Furthermore, unsupportive family members to follow healthy diet was a barrier; similar findings are also reported from studies conducted in Africa [23] and Portugal [74] and United States [75]. Family support is dependent on the relationship between people with diabetes and other family members, and employment status [63]. As with other studies from the Asia, Africa, United states, Europe we found an enabling factor to adhere to recommended self-management practices was support from friends and family through motivation [10, 19, 30, 64, 74, 76, 77], and keeping the company when practising diabetes self-management [31]. The family motivates people with diabetes through reminding them to take medicines and providing them financial assistance for medicines and visits to a doctor. Family support is needed to maintain diabetes self-management practices at home through reminders and emotional support [63].

Our results support findings elsewhere that peers/friends were supportive of diabetes self-management practices [32, 77, 78]. Shared of knowledge and experience of diabetes self-management practices among people with diabetes people helped them remain physically active. This was also reported in a previous study from Nepal [31].

Our finding that neighbours were sometimes barriers to diabetes self-management practices is consistent with previous study from the capital city of Nepal [30]. In Nepalese society, people share their issues including health problems to neighbours and in return neighbours provides their viewpoint on the treatment and management of health problems based on their knowledge and experience. This practice is fostered by a lack of sufficient counselling from physicians. One aspect of living in harmony in a community is having trust and respect to the neighbours. This might influence people to listen on health advice and follow it without evaluating the scientific value.

Insufficient support such as the lack and continuity of counselling from doctors were barriers to diabetes self-management practices. This finding corroborates a previous study from Bangladesh. People with diabetes might not understand the message completely from the health professionals due to lack of counselling in each visit. This, avoids concordance with diabetes self-management practices [79]. Two reviews from South Asia reported that people with diabetes rely on doctors for reliable information on diabetes management [60, 80]. However, given the increasing burden of diabetes and a low doctor to patient ratio (1:1429) in Nepal [37] it is unrealistic to expect such comprehensive counselling only from doctors. A review of 67 different countries reported an average consultation time of 5 min [81], insufficient for comprehensive counselling to people with diabetes.

In this study, inadequate information from low-level health care providers such as auxiliary health workers and health assistants on diabetes self-management practices was reported as a barrier to the provision of diabetes education. A possible explanation could be that only 17.4% of health care workers in Nepal received training on the orientation of NCD services [82]. There could be an opportunity to train lower level health care providers and nurses on diabetes education. Another option could be peer support programs and community-based programs to educate people with diabetes. Recently, female community health volunteers have seen the potential of counselling and screening for diabetes in Nepal [83].

A good relationship between people with diabetes and doctors was found to be an enabling factor for diabetes self-management practices, a finding also reported by previous studies [31, 34, 77]. This is not surprising as people with diabetes consider doctors as a credible source of information related to disease management, and they tend to trust and follow doctor’s advice [31].

### Community level factors

#### Cultural values

In line with existing literature [30, 60, 74] this study found that cultural practices that are a barrier to healthy dietary regimes include unhealthy food preparation styles, preferences for a carbohydrate diet, and festivals and social events at which food plays a significant cultural and social role, putting pressure on people with diabetes to abandon their healthy diet. Nepalese celebrate diverse festivals throughout the year; food preparation involves ghee (clarified butter), sugar, refined flours, and different sources of fat [30]. Therefore, any future interventions for management of diabetes should have both cultural and contextual understanding of major risk factors of diabetes, and factors that lead to poorer health outcomes among people with diabetes [84].

#### Availability of resources

Irregular supply of medicine in health facilities was a barrier to adhere to recommended medications. This supports a previous study that reported 20% of district hospital users and 40% of PHC users mentioned the unavailability of diabetes medicine all year round [85], adding further financial difficulty to people with diabetes who must buy medicine from private pharmacies [76]. A regular supply of medicines to hospitals and PHCs would resolve this barrier.

Recreational facilities in the community encourage people with diabetes to engage in and sustain regular physical exercise [63]. However, a lack of recreational public space is a barrier to physical activity, especially in urban areas, which was also reported in a study from Bangladesh [86]. Our finding that a locally available, nearby health facility was helpful for people with diabetes to visit when required has been recognised previously [10].

### Policy level factors

#### Lack of protocols and guidelines

This study reported that lack of protocols and guidelines for medical doctors to provide diabetes education was a barrier, which is supported by the previous study from Nepal reporting the lack of contextual guidelines for diabetes management [33]. In recent years, the Government of Nepal has emphasised NCDs management, though a significant process is yet to be made in the implementation [87]. For instance, in 2014, a multi-sectoral plan was adopted and a package for essential NCDs was launched [33], however that package does not include protocols for counselling on diabetes self-management practices. Our study found that it was essential to develop protocols that address contextual socio-cultural factors in order to improve practice. Another finding of this study was the need of culturally specific guidelines for diabetes self-management practices, also recommended by a previous review [60]. The Nepalese health system has several challenges such as inadequate budget allocation and lack of health insurance to cover diabetes care [33]. The availability of only one type of medicine at the district hospital and PHC level makes people with diabetes to buy other medication (including insulin) for diabetes at highly unaffordable price [88]. In addition, the provision of free diabetes medication and blood glucose tests in government health facilities was mentioned for consistent diabetes self-management practices in Nepal [33].

### Economic factors

The economic burden of the cost of medication, blood glucose monitoring, a healthy diet and appropriate footwear was a barrier to manage Type 2 diabetes. These findings were confirmed in a multi-national study [25] and other study conducted in Bangladeshi context [89]. Although this study did not explore socio-economic status of people with diabetes, economic issue also contributes to non-compliance of self-management practices. The explanation could be that out of pocket expenditure for healthcare costs in Nepal is 60%; people with diabetes are likely to pay for medical care [90]. Additionally, there is no insurance system in place. The public health facilities at a district level in Nepal offers free service for consultation with medical doctors but the associated costs such as laboratory tests, medicine and transportation costs are not covered. This situation adds additional economic strain on people with diabetes and their families. Additionally, patients find it difficult to regularly purchase healthy food because of increasing prices [33].

### Strengths and limitations of the study

This is one of the few studies that captured multiple stakeholders’ perspectives on barriers to, facilitators of diabetes self-management practices in Nepal. The triangulation of multiple views helped to identify barriers and facilitators to diabetes self-management practices, which can be used to initiate strategies to overcome barriers and reinforce facilitators. In addition, information on barriers, facilitators and improvement of diabetes self-management practices can be useful when developing programs to improve diabetes self-management knowledge, skills and practices of people with diabetes [91]. Data was collected in the Nepali language by a native speaker, which facilitated the emotions and perspectives to be captured without distortion. Finally, this study will serve as a baseline information for the future research of diabetes self-management practices in Nepal.

This study was subject to certain limitations. First, it included people with Type 2 diabetes who attended public health facilities and included doctors and district health managers who all belonged to government organisations. Like elsewhere in low and middle-income countries, public sector facilities are major gateway of health services for the management of diabetes among low socio-economic status patients in Nepal. Therefore, the findings cannot be easily generalised to people with diabetes who receive private care. Secondly, the findings should be interpreted with caution, as this study did not explore socioeconomic status of the people with diabetes. Future studies are needed to shed light upon such factors.

## Conclusion

People with Type 2 diabetes experience different types of barriers and facilitators to diabetes self-management practices. The important barriers were lack of knowledge of people with Type 2 diabetes about diabetes self-management practices, cultural practices related to diabetes self-management, insufficient counselling from doctors, lack of guidelines and protocols for counselling, lack of availability and accessibility of resources, and financial problems. The major facilitators were motivation to practice diabetes self-management, self-responsibility for disease management, support from family and peers, support from doctors, and the availability of resources in the community.

Some programmatic recommendations are suggested on the basis of this study. First, building patients’ knowledge and developing their skills will enable them to comply with and maintain recommended self-management practices [92]. Also, programs should be developed with an emphasis to improve self-efficacy of the people with diabetes to comply with diabetes management recommendations. Individuals and families under economic hardship and those who lack family support should receive better attention during design of future interventions.

Second, evidence based guidelines for health workers to educate or counsel people with diabetes on diabetes self-management practices is needed [93], as are and programs and guidelines for program managers (public health professionals and senior medical officers) to implement self-management packages. Third, understanding of issues for managing diabetes self-management from multiple actors is paramount as well as engaging multidisciplinary team for diabetes care and management [92]. Fourth recommendation is to train low-level health workers to provide diabetes self-management education to people with diabetes. Further, community awareness programs should be developed to increase knowledge about diabetes self-management practices among general population as well as people with diabetes. Finally, self-help support groups can be introduced to provide counselling in diabetes self-management practices and emotional support to people with diabetes.

## Supplementary Information

**Additional file 1.** Focus Group Discussion guide- People with Type 2 diabetes.

**Additional file 2.** Semi-structured guide- Caregivers.

**Additional file 3.** Semi-structured guide- Medical doctors.

**Additional file 4.** Semi-structured guide- District health managers/social worker.

## Data Availability

The transcript can be made available for the institution or Individual with special need or request based on case by case basis. The corresponding author will be able to provide data on reasonable request.

## Abbreviations

FGD: Focus group discussion
NCD: Non-communicable disease
PHC: Primary health care centre
SSI: Semi-structured interview
