# Supplementary material for: Barriers to and facilitators of diabetes self-management practices in Rupandehi, Nepal- multiple stakeholders’ perspective
Source: BMC Public Health. 2021 Jun 29;21:1269. doi: 10.1186/s12889-021-11308-4 (PMC8243465; doi:10.1186/s12889-021-11308-4)
Supplement: Supplementary file 1 — Additional file 1. Focus Group Discussion guide- People with Type 2 diabetes. [file 12889_2021_11308_MOESM1_ESM.docx]

**Focus Group Discussion guide- People with Type 2 diabetes**

Hello! My name is _________. I would like to welcome and thank you for volunteering to take part in this focus group. Today I would like to hear about your experiences on diabetes self-management. Then I will ask you some questions about the problems you face to manage your diabetes and the helping factors to manage your diabetes at home.

The focus group discussion will take no more than two hours. May I tape the discussion to facilitate its recollection? (If yes, switch on the recorder)

Despite being taped, I would like to assure you that the discussion will be anonymous. The tapes will be kept safely in a locked facility until they are transcribed word for word, then they will be destroyed. The transcribed notes of the focus group will contain no information that would allow individual subjects to be linked to specific statements. You should try to answer and comment as accurately and truthfully as possible. I and the other focus group participants would appreciate it if you would refrain from discussing the comments of other group members outside the focus group. If there are any questions or discussions that you do not wish to answer or participate in, you do not have to do so; however please try to answer and be as involved as possible.

**Ground rules**

- The most important rule is that only one person speaks at a time. There may be a temptation to join in when someone is talking, but please wait until they have finished.
- There are no right or wrong answers
- You do not have to speak in any particular order
- When you do have something to say, please do so. There are many of you in the group and it is important that I obtain the views of each of you
- You do not have to agree with the views of other people in the group
- Does anyone have any questions? (answers).

OK, let’s begin

Date………………..

No of participants……………

**Warm up**

- First, I’d like everyone to introduce themselves. Can you tell us your name?
- Can you provide me demographic information? (fill in another form)

**Introductory question**

- I am just going to give you a couple of minutes to think about your experience of living with diabetes. Is anyone happy to share his or her experience?
- **Guiding questions**

1. How is your overall experience with managing diabetes over the past year?

- Probes: Diet and nutrition, physical activity,taking medication,blood glucose monitoring, foot care

1. Have you encountered any problems to manage your diabetes (Diet and nutrition, physical activity, taking medication, blood glucose monitoring and foot care)? What are they?

- Probes: intrapersonal (knowledge, motivation, responsibility), interpersonal (relationship with family, friends/peers, health professionals, and neighbours), institutional (health system factors), community (cultural values, availability and accessibility of resources for diabetes self-management practices) and public policy factors (diabetes self-management practices policies and funding)

1. Can you describe how the problems/difficulties prevents you from managing your diabetes?

- Probes: intrapersonal (knowledge, motivation, responsibility), interpersonal (relationship with family, friends/peers, health professionals, and neighbours), institutional (health system factors), community (cultural values, availability and accessibility of resources for diabetes self-management practices) and public policy factors (diabetes self-management practices policies and funding)

1. What are the helping factors for you to manage your diabetes? Please describe how they helped you to manage your diabetes.

- Probes: intrapersonal (knowledge, motivation, responsibility), interpersonal (relationship with family, friends/peers, health professionals, and neighbours), institutional (health system factors), community (cultural values, availability and accessibility of resources for diabetes self-management practices) and public policy factors (diabetes self-management practices policies and funding)

**Closing**

Summarize the general themes of the FGD and ask:

- Is this a good representation of what was said?
- Does anyone else have anything to add?

**Conclusion**

- Thank you for participating. This has been a very successful discussion
- Your opinions will be a valuable asset to the study
- We hope you have found the discussion interesting
- If there is anything you are unhappy with or wish to complain about, please speak to me later
- I would like to remind you that any comments featuring in this report will be anonymous
