# Supplementary material for: Barriers to and facilitators of diabetes self-management practices in Rupandehi, Nepal- multiple stakeholders’ perspective
Source: BMC Public Health. 2021 Jun 29;21:1269. doi: 10.1186/s12889-021-11308-4 (PMC8243465; doi:10.1186/s12889-021-11308-4)
Supplement: Supplementary file 4 — Additional file 4. Semi-structured guide- District health managers/social worker. [file 12889_2021_11308_MOESM4_ESM.docx]

**Semi-structured guide- District health managers/social worker**

Hello! My name is ___________ Today I would like to hear about your opinions on the problems/issues and enablers experienced by the people with type 2 diabetes to perform self-management of diabetes.

1. Sex Male☐ Female ☐
2. Name of Respondent: ___________________________________
3. What do you think are the difficulties for the people with type 2 diabetes to manage diabetes? Please describe.
4. What do you think are the difficulties for the people with type 2 diabetes to manage diabetes? Please describe.

Probes

- intrapersonal (knowledge, motivation, responsibility),
- interpersonal (relationship with family, friends/peers, health professionals, and neighbours),
- institutional (health system factors), community (cultural values, availability and accessibility of resources for diabetes self-management practices)
- public policy factors (diabetes self-management practices policies and funding)

1. What do you think are the facilitaters for the people with type 2 diabetes to manage diabetes? Please describe.

Probes

- intrapersonal (knowledge, motivation, responsibility),
- interpersonal (relationship with family, friends/peers, health professionals, and neighbours),
- institutional (health system factors), community (cultural values, availability and accessibility of resources for diabetes self-management practices)
- public policy factors (diabetes self-management practices policies and funding)

1. Do you have anything to add more?
